# Enhanced legume growth and adaptation to degraded estuarine soils using *Pseudomonas* sp. nodule endophytes

**DOI:** 10.3389/fmicb.2022.1005458

**Published:** 2022-10-20

**Authors:** Noris J. Flores-Duarte, Sara Caballero-Delgado, Eloisa Pajuelo, Enrique Mateos-Naranjo, Susana Redondo-Gómez, Salvadora Navarro-Torre, Ignacio D. Rodríguez-Llorente

**Affiliations:** ^1^Department of Microbiology and Parasitology, Faculty of Pharmacy, University of Sevilla, Sevilla, Spain; ^2^Department of Plant Biology and Ecology, Faculty of Biology, University of Sevilla, Sevilla, Spain

**Keywords:** contaminated estuarine soils, enhanced nodulation, nodule endophytes, plant growth-promoting bacteria, phytostabilization

## Abstract

The joint estuary of Tinto and Odiel rivers (SW Spain) is one of the most degraded and polluted areas in the world and its recovery is mandatory. Legumes and their associated bacteria are recommended sustainable tools to fight against soils degradation and loss of fertility due to their known positive impacts on soils. The aim of this work was to isolate and characterize plant growth promoting nodule endophytes (PGPNE) from inside nodules of *Medicago* spp. naturally growing in the estuary of the Tinto and Odiel Rivers and evaluate their ability to promote legume adaptation in degraded soils. The best rhizobia and non-rhizobia among 33 endophytes were selected based on their plant growth promoting properties and bacterial enzymatic activities. These strains, identified as *Pseudomonas* sp. N4, *Pseudomonas* sp. N8, *Ensifer* sp. N10 and *Ensifer* sp. N12, were used for *in vitro* studies using *Medicago sativa* plants. The effects of individual or combined inoculation on seed germination, plant growth and nodulation were studied, both on plates and pots containing nutrient-poor soils and moderately contaminated with metals/loids from the estuary. In general, inoculation with combinations of rhizobia and *Pseudomonas* increased plant biomass (up to 1.5-fold) and nodules number (up to 2-fold) compared to single inoculation with rhizobia, ameliorating the physiological state of the plants and helping to regulate plant stress mechanisms. The greatest benefits were observed in plants inoculated with the consortium containing the four strains. In addition, combined inoculation with *Ensifer* and *Pseudomonas* increased As and metals accumulation in plant roots, without significant differences in shoot metal accumulation. These results suggest that PGPNE are useful biotools to promote legume growth and phytostabilization potential in nutrient-poor and/or metals contaminated estuarine soils.

## Introduction

Industrial and mining activity releases toxic metal/loids into the environment, such as As, Cd, Cu, and Pb, all of them very harmful to human health and most living beings (FAO and ITPS, 2015). In turn this has caused the degradation of soils, either by the modification of pH or by originating a decrease in the number of arable lands causing a problem at social, economic, and environmental levels (Singh et al., 2010; Chen et al., 2013). Heavy metals are dangerous because they are not degradable either chemically or biologically, being able to remain in the environment for hundreds or thousands of years (Herrera Marcano, 2011). These metals have also been repressing the enzymatic activity of the soils, causing a decrease in the growth and respiration of the populations of microorganisms, thus altering the diversity present in the rhizosphere (Abdu et al., 2017; Aponte et al., 2020). Marshes of river estuaries are particularly sensitive to heavy metals contamination by metals deposition. A good example is the combined estuary of Tinto and Odiel rivers (Huelva, SW Spain), known as one of the most contaminated regions in the world (Hudson-Edwards et al., 1999). Levels of toxic metal/loids as high as 125 ppm of As, 890 ppm of Cu, 275 ppm of Pb or 1,500 ppm of Zn in the Odiel river marshes, and 2 to 3-fold these levels in the Tinto River marshes, have been reported in the last decade (Mesa et al., 2015a; Navarro-Torre et al., 2017). These levels exceed those allowed by regional and national legislation in natural parks, agricultural and industrial soils (Junta de Andalucía and Consejeria de Medio Ambiente, 1999).

One solution to this great environmental problem is the implantation of pioneering crops able to regenerate the soil and improve its yield, respecting the environment. This kind of strategies should include the use of leguminous plants (belonging to the *Fabaceae* family), due to their great benefits. First, legumes are an important product in the food industry both for human and animal consumption worldwide, since they are very rich in protein, fiber, vitamins, and minerals and also has a low cost, being highly consumed in underdeveloped countries (Ferreira et al., 2021). Second, legumes play a very important role in agriculture as cover crops, since they improve soil fertility increasing crops yield, help the constant movement of soil nutrients and release matter into the soil (Stagnari et al., 2017; Ferreira et al., 2021). Third, legumes are good candidates to adapt to degraded soils affected by biotic and abiotic factors, particularly these plants play an important role in the regeneration of soils degraded by heavy metals due to their ability to accumulate metals in the roots without affecting plant growth (Mesa et al., 2015b; Flores-Duarte et al., 2022a).

Legumes are capable of fixing atmospheric nitrogen through symbiosis with many genera of soil nitrogen-fixing bacteria, called rhizobia, which penetrate the roots through the root hairs, forming nodules in which nitrogen fixation takes place (Poole et al., 2018). But nodules are not only occupied by rhizobia, within the legume nodules there are another great variety of bacteria called non-rhizobial nodule-associated bacteria (NAB; Rajendran et al., 2012), non-rhizobial endophytes (NRE; De Meyer et al., 2015), or just nodule endophytes (Velázquez et al., 2013). These bacteria do not induce nodule formation but can colonize nodules accompanying rhizobia forming a beneficial association and enhancing nodulation and plant growth. Within this group, bacteria of the genera, *Acinetobacter*, *Agrobacterium*, *Bacillus, Burkholderia, Pseudomonas* and *Variovorax*, among others, have been found (Shiraishi et al., 2010; Martínez-Hidalgo and Hirsch, 2017; Bessadok et al., 2020). These nodule endophytes usually behave as plant growth promoting bacteria (PGPB).

Soil health and fertility are directly influenced by beneficial plant-microbe relationships that determine soil biodiversity (Vishwakarma et al., 2020). Plant-PGPB interactions have shown to provide benefits in plant growth and development by facilitating the acquisition of nutrients (Fasusi et al., 2021) and producing phytohormones related with plant and root lenth growth, and the formation of root hairs and lateral roots, such as cytokinin, gibberellins, abscisic acid, and IAA (Raza et al., 2019). Through atmospheric nitrogen fixation (Roy et al., 2020), phosphate mobilization/solubilization (Etesami et al., 2021) and siderophores production (Lurthy et al., 2020; Kang et al., 2021) bacteria provide N, P, Fe, and Zn to plants. PGPB also play a determinant role in plant adaptation and tolerance to biotic and abiotic stress (Chaudhary et al., 2022). For example, bacteria with ACC-deaminase activity are able to break the ethylene precursor ACC, altering plant stress perception (Penrose and Glick, 2003; Chandwani and Amaresan, 2022).

Microorganisms have an important role in pollutant detoxification and heavy metal plant stress resistance (Caracciolo and Terenzi, 2021). Soil microorganisms have developed different resistance mechanisms, such as metal biosorption, bioaccumulation, modification of metal chemical state (methylation) or production of chelating compounds, particularly siderophores and biosurfactans, that cause a lowering in metal availability for plants (Verma and Kuila, 2019), then diminishing plant metal content (Caracciolo and Terenzi, 2021). PGPB are directly involved in metal detoxification through the production of secondary metabolites, such as siderophores, ACC or IAA (Caracciolo and Terenzi, 2021). Particularly in legumes, nitrogen-fixing bacteria and endophytes inside nodules have probed to reduce metal translocation to aerial parts and increase plant nitrogen content and growth in metal contaminated soils (Navarro-Torre et al., 2020; Flores-Duarte et al., 2022b).

In a recent work, a consortium of PGPB, including *Pseudomonas*, *Chryseobacterium* and *Priestia* genera, showed their ability to promote *Medicago sativa* growth and adaptation in poor-nutrient soils (Flores-Duarte et al., 2022b). These bacteria were isolated from the rhizosphere of different legumes growing in estuarine soils with low levels of minerals and organic matter. It is well accepted that endophytes maintain a much closer relationship with the plant and have lower competition with soil microorganisms than rizospheric bacteria (Adeleke and Babalola, 2021; Dwibedi et al., 2022). The present work is aimed to determine whether a consortium of endophytes isolated from nodules could enhance legume growth in degraded soils more efficiently than rhizosphere bacteria, introducing soil metal contamination as additional stress.

The objectives that arise in this work are the following: (i) isolation and characterization of rhizobia and endophytes from nodules of *Medicago* spp. naturally growing in the Odiel river estuary (Huelva, Spain); (ii) selection of rhizobia and nodule endophytes based on their properties, enzymatic activities and ability to tolerate metal/loids; (iii) determine the ability of the selected bacteria to promote *M. sativa* growth, nodulation and metal accumulation in Odiel marshes soils under greenhouse conditions.

## Materials and methods

### Collection of samples and soil characterization

Nodulated wild plants of *Medicago* spp. were collected from high marshes of the Odiel river estuary (Huelva, Spain; 37°150 N, 6°580 W; SW Spain) in February 2020. Soil samples (15–20 cm deep) were collected using a shovel, gloves and plastic bags. The samples were immediately transported to the laboratory and stored at 4°C. Three homogeneous soil samples were deposited in sterile bottles for chemical soil analysis, as described by Mateos-Naranjo et al. (2015). The soil texture (percentage of sand, silt, and clay) was determined using the Bouyoucos hydrometer method (Bouyoucos, 1936). Electrical conductivity was measured with a Crison-522 conductivity meter (Spain) and pH and redox potential with a Crison pH/mVp-506 portable meter (Spain). The concentration of nutrients in the soil was measured by inductively coupled plasma-optical emission spectroscopy (ICP-OES; ARLFisons3410, Thermo Scientific, Walthman, MA, United States). The amount of organic matter was determined by the method of Walkley and Black (1934). Results are presented in Table 1.

**Table 1:** Physicochemical properties and micronutrients concentrations of soil from high marshes of the Odiel River.

Physicochemical properties									
Location		Texture (%) *		Organic material (%)		Conductivity (μS·cm-^1^)		pH	
High marsh		72/13/15		0.9 ± 0.01		13.1 ± 0.3		6.8 ± 0.2	
**Metal/loid concentration (mg·kg^−1^)**									
**Location**		**As**	**Cd**	**Cu**	**Zn**	**Mg**	**Na**	**Fe**	**P**
High marsh		27.9 ± 2.2	0.38 ± 0.01	316.7 ± 3.20	345.0 ± 7.10	0.789 ± 0.01	0.369 ± 0.01	9345.56 ± 9.35	0.056 ± 0.01

Values are mean ± S.D. (*n* = 3).

* Texture (sand/slit/clay percentage).

### Isolation of endophytic bacteria from nodules of *Medicago* spp.

Nodules were exscinded from roots with a scalpel, washed with water and placed on tubes. Samples were immersed in 70% (v/v) ethanol for 1 min with manual agitation to disinfect the surface. Then, they were washed again with sterile water and immersed in 3% (v/v) sodium hypochlorite for 15 min under continuous agitation and finally washed four times with sterile distilled water. Disinfected nodules were deposited in a mortar with 1 ml of sterile 0.9% (w/v) saline solution and grinded. Portions of 100 μl of the resulting mix were to spread and extended with a spatula in Petri dishes with TY medium (tryptone-yeast extract agar) and TSA (tryptone soya agar, Intron Biotechnology, Korea). Plates were incubated for 72 h at 28°C. Controls of nodule surface disinfection and solution sterility were performed. Bacteria were separated based on the different colony morphology and cell morphology was observed by Gram stain using an Olympus CX41 microscope with the 100x objective.

### Determination of plant growth promoting properties and enzymatic activities

The ACC deaminase activity was assessed as described by Penrose and Glick (2003) and adapted by Mesa et al. (2015a). Activity was measured by monitoring α-ketobutyric acid at 540 nm in a spectrophotometer (Lambda25; PerkinElmer, Walthmam, MA, United States), and the amount of α-ketobutyric acid was determined using a standard curve with known concentrations. ACC deaminase activity was expressed in μmoles of α-ketobutyrate per mg of protein per hour. To verify nitrogen fixation, strains were plated in nitrogen free broth (NFB; Döebereiner, 1995) for 5 days at 28°C. Indole-3-acetic acid (IAA) production was determined using a colorimetric technique by incubating 3 ml of a liquid culture of TY or TSB supplemented with L-tryptophan (0.1 mg/ml) and incubated at 28°C for 72 h at 200 rpm. Cultures were then centrifuged for 5 min at 13,000 rpm and supernatants transferred to glass tubes. The appearance of a pink color after adding 4 ml of Salkowski’s reagent (Gordon and Weber, 1951) indicated that the test was positive. The amount of IAA was calculated by measuring the absorbance at 535 nm in a spectrophotometer (Lambda 25; Perkin Elmer, Waltham, MA, USA). Phosphate solubilization was carried out on plates with NBRIP medium (phosphate growth medium from the National Institute of Botanical Research) as described by Nautiyal (1999). The appearance of a transparent halo after 7 days of incubation at 28°C indicated that the bacteria had the capacity to solubilize phosphates. 100 μl of bacterial culture medium was added to each well. The formation of biofilms will be prolonged by checking the adhesion capacity of the bacteria in microplates with 96 wells in TY or liquid TSB at 28°C, incubating for 4 days, after incubation each well was stained with 200 μl of 0.01% crystal violet as described by del Castillo et al. (2012). Siderophores production was determined by the appearance of an orange halo around the well containing 100 μl of bacterial culture in CAS medium (Chromeazurol S), after incubation for 7 days at 28°C (Schwyn and Neilands, 1987). Enzymatic activities were determined on plates incubated at 28°C for 7 days. Pectinase and cellulase activities were examined as described by Elbeltagy et al. (2000). For pectinase activity, strains were plated on ammonium mineral agar (AMA). Plates were revealed with 2% CTAB and positive bacteria showed a halo around. For cellulase activity, strains were plated on solid M9 minimal medium supplemented with yeast extract (0.2%) and carboxymethylcellulose (1%). Plates were developed by covering the plate with 1 mg/ml Congo Red solution for 15 min and decolorizing with 1 M NaCl for 15 min. The appearance of a clear halo indicated positive result. Chitinase activity was performed as described by Mesa et al. (2015a). Amylase activity was performed on starch agar plates (Scharlab, Barcelona, Spain) and revealed with 10 ml lugol. The formation of a transparent halo indicated positive result. Lipase and protease activities were observed by the presence of halos around the bacteria after incubation in casein agar and Tween 80 mediums, respectively, as described by Prescott (2002). And finally, the DNAse activity was determined by cultures on DNA agar plates revealed with 1 M HCl.

For PGP properties and enzyme activities, positive and negative controls from BIO-181 group collection were used (Supplementary Table 1).

### Tolerance to metals/loids

The isolated bacteria were plated in TY and TSA medium with increasing concentrations of As, Cd, Cu and Zn, from 1 M NaAsO_2_, 1 M CdCl_2_, 1 M CuSO_4_, 1 M ZnSO_4_ stock solutions. Plates were incubated for 24–48 h at 28°C and tolerance expressed as the maximum tolerable concentration (MTC), that is the maximum concentration that allows visible bacterial growth.

### Analysis of diversity and identification of isolates

The diversity of the isolated endophytes was analyzed by performing a Box-PCR. Bacterial genomic DNA was isolated using a G-spin™ Genomic DNA Extraction Kit for Bacteria (Intron Biotechnology, Gyeonggi-do, Korea) with the instructions determined by the manufacturer. Box-PCR was performed using 1 μl of DNA and Box A1R primer (5′-CTACGGCAAG GCGACGCTGACG-3′) using the Maxime™ PCR PreMix kit (i-Taq™; Intron Biotechnology, Gyeonggi-do, Korea) and following the PCR conditions: initial denaturation at 94°C for 2 min, 30 cycles of denaturation at 94°C for 20 s, annealing at 52°C for 20 s, extension at 72°C for 1 min, and final extension at 72°C for 5 min. Electrophoresis was performed in a 1.5% (w/v) agarose gel and a voltage of 70 V for 2 h. Representative bacteria of each different Box-PCR profile were identified by 16S rRNA gene amplification using 16F27 and 16R1488 primers (Navarro-Torre et al., 2016) and the Maxime™ PCR PreMix kit (i-Taq™; Intron Biotechnology, Gyeonggi-do, Korea) following the next PCR conditions: initial denaturation at 94°C for 2 min, 30 cycles of denaturation at 94°C for 20 s, annealing at 58°C for 10 s, extension at 72°C for 50 s, and final extension at 72°C for 5 min. Electrophoresis was performed in a 1% (w/v) agarose gel and a voltage of 120 V for 30 min. PCR products were purified with the enzyme ExoSAP IT (Affymetrix, Santa Clara, CA, USA), following the manufacturer instructions, and sequenced by the StabVida company (Caparica, Portugal). Then, 16S rRNA gene sequences were compared with those deposited in the EzBioCloud database (Yoon et al., 2017) using the Ez-Taxon e service (www.ezbiocloud.net/eztaxon; accessed July 2022). Finally, 16S rRNA gene sequences were deposited in the NCBI GenBank.

### Inoculant design

Selected bacteria were grown together on TY plates to test growth compatibility as described in Navarro-Torre et al. (2016). The inoculants were prepared by cultivating selected strains separately in TSB or TY at 28°C for 24 h and 200 rpm. Afterwards, cultures containing 10^8^ cells mL^−1^ were transferred to sterile Falcon tubes to be centrifuged at 8000 rpm for 10 min, washing twice with sterile 0.9% (w/v) saline solution, to remove traces of the culture medium (Navarro-Torre et al., 2016). At the end of the washes the cultures were resuspended in a sterile liquid nitrogen deficient buffered nodulation medium (BNM; Ehrhardt et al., 1992), or sterile water for experiments under greenhouse conditions. For co-inoculation and consortium inoculation designs, bacteria were mixed after resuspension. Inoculation conditions were defined as follow: C-: non inoculation; N4: inoculation with *Pseudomonas* sp. N4; N8: inoculation with *Pseudomonas* sp. N8; N10: inoculation with *Ensifer* sp. N10; N12: inoculation with *Ensifer* sp. N12; N4 + N10: co-inoculation with *Pseudomonas* sp. N4 and *Ensifer* sp. N10; N8 + N10: co-inoculation with *Pseudomonas* sp. N8 and *Ensifer sp.* N10; N4 + N12: co-inoculation with *Pseudomonas* sp. N4 and *Ensifer* sp. N12; N8 + N12: co-inoculation with *Pseudomonas* sp. N8 and *Ensifer sp.* N12; CSN: inoculation with *Pseudomonas* sp. N4*, Pseudomonas* sp., N8 *Ensifer* sp. N10 and *Ensifer* sp. N12.

### Labelling of *Pseudomonas* with fluorescence and microscopy

*Pseudomonas* strains (*Pseudomonas* sp. N4 and *Pseudomonas* sp. N8) were marked with the fluorescent protein mCherry by triparental matting, mixing the donor *Escherichia coli* DH5α containing the plasmid pMP7604 (Lagendijk et al., 2010), the helper strain *E. coli* containing pRK600 (Finan et al., 1986) and *Pseudomonas* strains spontaneously resistant to rifampicin (100 μg/ml) as recipients. 100 μl aliquots of TSB overnight cultures were plated on TSA containing rifampicin (100 μg/ml) and tetracycline (10 μg/ml). Fluorescent *Pseudomonas* resistant to both antibiotics thus containing the plasmid were selected. *M. sativa* plants were co-inoculated with the rhizobia (*Ensifer* sp. N10 or *Ensifer* sp. N12) and labelled *Pseudomonas* in square plates (12 × 12 cm), 20 seedlings per condition, as described below. After 28 days, roots and nodules were cut with a sterile scalpel and 0.5 mm cuts observed (Mesa et al., 2015b). Fluorescent bacteria in plant tissues were visualized using a laser scanning spectral confocal optical microscope (Zeiss LSM 7 DUO, Zeiss, Jena, Germany) with an objective Plan-Apochromat 20X/0.8 M27, filters of 572–727, and a laser of 561 nm (5.3%). Images were processed with ZEN2011 software (Zeiss, Jena, Germany).

### *Medicago sativa* seeds germination and nodulation on plates

Alfalfa (*M. sativa*) seeds were surface disinfected following the protocol described by Navarro-Torre et al. (2019) by immersing them for 10 min in 70% (v/v) ethanol, followed by 30 min in sodium hypochlorite at 3% (v/v) under gentle agitation, washing 6 times with sterile distilled water. The next step was to immerse 50 seeds in each inoculant for 1 h and for the non-inoculated control (C-) seeds were immersed for 1 h in 0.9% (w/v) NaCl. Seeds were transferred to plates containing 0.9% (w/v) water-agar (5 repetitions per condition and 10 seeds per plate) or water-agar plates containing a mixture of 7.5 μM As, Cd, Cu, and Zn, prepared from the stock solutions described above (paragraph 2.4), and finally incubated in the dark at 28°C. Germination was observed every 24 h for 7 days in absence of metals and for 21 days in presence of metals.

For *in vitro* nodulation experiment, pre-germinated seeds were transferred to square plates (12×12 cm) with nitrogen-free BNM-agar medium (Ehrhardt et al., 1992), supplemented with sodium arsenite at a concentration of 30 μM. or in absence of arsenite. NH_4_NO_3_ was added to all plates at a concentration of 1 mM. 10 seeds were deposited in each plate and 5 replicates per treatment were performed. The seeds were inoculated or co-inoculated under the conditions described above using the selected bacteria, and non-inoculated seeds were used as controls. The plates were placed in a vertical position, incubated at 22°C with a cycle of 8 h of darkness and 16 h of light (120–130 μE m ^−2^·s^−1^) in a growth chamber (AGP-700-HR ESP; Radiber, Barcelona, Spain). Plants were harvested after 21 days and nodules from each condition were counted.

### Experiments under greenhouse conditions

Soil was collected from the upper marshes of the Odiel River and sterilized in an autoclave at 121°C and 1 atm of overpressure for 30 min. This sterilization was repeated twice. To confirm soil sterilization, 3 samples of 1 g of soil were washed by shaking in 10 ml of sterile water. After decanting for 10 min, three samples of 100 μl of supernatant per tube were plated on TSA and no growth was observed on plates incubated 48 h at 28°C. Plastic pots (11 cm squared pots with 12 cm height) were filled with 1 kg of the previously sterilized soil (3,1, three parts of the soil from the marshes and one part of sterile perlite) and 2 pre-germinated seeds were placed per pot, 8 pots per condition. The experiment lasted 60 days and plants were irrigated with sterile water once a week (50 ml) and weekly inoculated with their respective inoculum (50 ml of cultures). The greenhouse had controlled light and temperature conditions; natural light was supplemented with fluorescent/incandescent lamps to get a photoperiod of 16 h light: 8 h dark; and the temperature was adjusted to 25°C during the day and 15°C during the night. At the end, the length of roots and shoots were measured, the number of nodules and leaves were counted, the diameters of the leaves were determined, and the nitrogen content in the plant was evaluated using an InfrAlyzer 300 (Technicon, Tarrytown, NY, United States), as described by Carrasco et al. (2005). Finally, samples were placed in an oven at 80°C for 48 h to determine the dry weight of both shoots and roots separately.

### Determination of photosynthetic parameters and total chlorophyll content

For the determination of gas exchange, leaves were randomly selected from *M. sativa* plants and measured with an infrared gas analyzer (IRGA) LI-6400 (LI-COR Biosciences, Lincoln, NE, USA) equipped with a Li-6,400-02B leaf light chamber. Measurements were made between 9 am and 2 pm under a photosynthetic photon flux density of 1,500 μmol m^−2^ s^−1^, a vapor pressure deficit of 2–3 kPa, at a temperature of approximately 25°C and an environment of CO_2_ concentration of 400 μmol·mol^−1^ air. Gas exchange stabilization (120 s) was equilibrated, and measurements were recorded to determine the net photosynthetic rate (A_N_). In addition, a fluorometric analysis was performed to study the energy use efficiency of photosystem II (PSII). The maximum quantum efficiency of PSII photochemistry (Fv/Fm) and the quantum efficiency of PSII (Φ_PSII_) were determined using a saturation pulse method described in Genty et al. (1989). Selected leaves were light- and dark-adapted for 30 min, followed by a saturated actinic light pulse of 10,000 μmol m^−2^ s^−1^ for 0.8 s at noon (1700 μmol photons m^−2^ s^−1^) using a FMS-2 modulated fluorimeter (Hansatech Instruments Ltd., Pentney, United Kingdom). Details are described in Schreiber et al. (1986). With the recorded data, the electron transport rate (ETR) was calculated as described in Krall and Edwards (1992). Finally, the total content of chlorophyll described by Hiscox and Israelstam (1979) was determined. 50 mg of randomly selected leaves were ground using a mortar containing 100% acetone: 0.9% saline solution (4.1; v/v). The resulting extract was resuspended in acetone and measured at 652 nm using a spectrophotometer (Lambda 25; PerkinElmer, Walthmam, MA, United States) and the total chlorophyll content was determined using the formula described by Arnon (1949). Samples were run in duplicate.

### Antioxidant enzyme analysis

Guaiacol peroxidase (GPX), catalase (CAT), superoxide dismutase (SOD) and ascorbate peroxidase (APX) enzymatic activity were measured with leaves and roots randomly collected from each of the treatments at the end of the experiment. Immediately after the collection, the samples were deposited in nitrogen liquid and stored at −80°C until homogenized. 48 h after storage, 500 mg of plant material were homogenized in extraction buffer (50 mM sodium phosphate buffer at pH 7.6) and centrifuged at 4°C and 9,000 rpm for 20 min, and the plant extract was stored at −80°C. Total protein concentration was measured in the plant extract following the Bradford method (Bradford, 1976) using a BSA standard curve. In guaiacol peroxidase activity (GPX), guaiacol oxidation was measured at 470 nm. Catalase (CAT) was determined by measuring the disappearance of H_2_O_2_ at 240 nm. Superoxide dismutase (SOD) activity was assayed using autoxidation of pyrogallol at 325 nm. Finally, for ascorbate peroxidase (APX), the oxidation of L-ascorbate was measured at 290 nm. For the determination of the autoxidation of substrates, control tests were carried out without the presence of the enzyme extract. Enzymatic activities were expressed as units per μg of protein and were measured in triplicate.

### Statistical analysis

Statistical analyzes were determined using Statistical version 12.0 software (Statsoft Inc., Tulsa, OK, United States). The normality of the results was verified using the Kolmogorov Smirnov test. The results of each treatment were compared using one-way ANOVA, and Fisher test was performed to determine statistical differences.

## Results

### Isolation and characterization of endophytic bacteria from nodules

#### Soil characterization

Bacteria were isolated from nodules of *Medicago* spp. that grows naturally in the high marshes of the Odiel River. The soil was composed of 72% sand, 0.9% organic matter, had low electrical conductivity and nutrients content, as well as moderate amounts of As, Cd, Cu and Zn. The soil characteristics are presented in Table 1.

#### Isolation of endophytes

A total of 33 endophytic bacteria were isolated from *Medicago* spp. nodules (13 strains grew in TY and 20 in TSA). They were identified based on colony morphology (color and shape of the colonies), cellular morphology (cocci, bacilli, coccobacilli) and type of cell wall by Gram staining (Gram positive or Gram negative). Other aspects such as grouping, if any (clusters, chains), or the presence of spores (central or terminal) were also observed. 85% of the strains were Gram negative bacilli and 15% were Gram positive bacilli, and only one of them presented spores (Supplementary Table 2). Subsequently, a Box-PCR of the 33 strains was performed in order to study the collection genotypically (Supplementary Figure 1). Several of the isolates showed identical band profiles, so the study was reduced to 24 strains for which the 16S rRNA genes were partially sequenced. The sequences showed similarity with *Achromobacter*, *Bacillus*, *Enterobacter*, *Ensifer, Lelliottia, Priestia* and *Pseudomonas* genera, being *Pseudomonas* and *Lelliottia* the most represented (Table 2).

**Table 2:** Identification of cultivable entophytic bacteria isolated from nodules of *Medicago* spp. using the EzBiocloud database.

Strain	Sequence size (bp)	Related species	% ID	Accession number
N1	461	*Achromobacter piechaudii*	99.57	OP060607
N2	602	*Lelliottia jeotgali*	96.75	OP060608
N3	343	*Lelliottia jeotgali*	95.27	OP060609
**N4**	**1,154**	**Pseudomonas mucoides**	**97.47**	**OP060610**
N5	722	*Lelliottia jeotgali*	98.53	OP060611
N6	1,218	*Achromobacter spanius*	99.01	OP060612
N7	1,147	*Lelliottia jeotgali*	98.27	OP060613
**N8**	**1,127**	**Pseudomonas mucoides**	**99.13**	**OP060614**
N9	694	*Lelliottia jeotgali*	99.54	OP060615
**N10**	**1,111**	**Ensifer meliloti**	**98.83**	**OP060616**
N11	1,126	*Pseudomonas mucoides*	98.84	OP060617
**N12**	**863**	**Ensifer meliloti**	**99.88**	**OP060618**
N13	1,051	*Lelliottia amnigena*	97.71	OP060619
N14	865	*Priestia megaterium*	99.65	OP060620
N15	1,310	*Pseudomonas lactis*	97.10	OP060621
N16	1,035	*Pseudomonas lactis*	99.13	OP060622
N18	1,374	*Bacillus velezensis*	96.44	OP060623
N20	1,328	*Pantoea vagans*	95.41	OP060624
N21	678	*Pseudomonas lactis*	99.26	OP060625
N23	949	*Enterobacter huaxiensis*	96.63	OP060626
N25	1,257	*Bacillus proteolyticus*	95.83	OP060627
N30	1,314	*Pseudomonas capeferrum*	96.12	OP060628
N32	950	*Bacillus tequilensis*	99.58	OP060629
N33	1,209	*Pseudomonas capeferrum*	98.76	OP060630

Selected strains appear in bold.

#### Characterization of PGP properties, enzymatic activities and metals tolerance

PGP properties of the endophytes were determined, showing all of them four to five out of six analyzed properties, while two strains presented the six properties (Table 3; Supplementary Table 3). All the bacteria were able to produce siderophores and most of them (30) solubilized phosphate (Supplementary Table 3). N4 strain showed the highest production of siderophores according to the diameter of the halo in CAS medium (Table 3; Supplementary Table 3) and N15, N20, N30 and N31 were the best phosphate solubilizers (Supplementary Table 3). A high number of strains were able to grow in N free medium (30 isolates) and/or produced auxins (27 isolates). N8 strain produced the highest concentration of IAA, close to 18 mg^.^L ^−1^ (Table 3; Supplementary Table 3). 20 strains formed biofilm and 11 isolates showed ACC deaminase activity, presenting N8 strain the highest activity, close to 10 μmol α-ketobutyrate mg protein^−1^ h^−1^ (Table 3; Supplementary Table 3).

**Table 3:** PGP properties and enzymatic activities showed by selected strains.

PGP properties	N4	N8	N10	N12
Phosphate solubilisation	11	12	15	14
Siderophores production	73	44	13	10.2
IAA production	1.562	17.939	1.266	1.00
Biofilm formation	+	+	+	+
N fixation	**+**	**+**	**+**	**+**
ACC deaminase activity	9.677	9.987	**−**	−
**Enzymatic activities**				
DNAse	−	−	+	+
Amylase	−	−	−	−
Cellulase	−	+	+	+
Lipase	−	−	−	−
Pectinase	−	−	−	−
Protease	+	+	−	−
Chitinase	−	−	−	−

N4: *Pseudomonas* sp. N4; N8: *Pseudomonas* sp. N8; N10: *Ensifer* sp. N10; N12: *Ensifer* sp. N12; +, presence of the activity; −, absence of the activity. Values of phosphate solubilisation and siderophores production express the diameter of the halo in mm. Values of IAA production are expressed in mg·L^−1^. Values of ACC deaminase activity are expressed in μmoles α-ketobutyrate·mg protein^−1^·h^−1^.

The presence of enzymatic activities in the endophytes was studied (Supplementary Table 4). Cellulase and protease activities were the most abundant, since they were found in 22 and 17 strains, respectively (Supplementary Table 3). A low number of isolates (2 to 5) showed pectinase, lipase, chitinase or DNase activities (Supplementary Table 4).

Tolerance of the strains towards As, Cd, Cu, and Zn were determined and expressed as the maximum tolerable concentration (Table 4; Supplementary Table 5). Endophytes showed good levels of metal/loids tolerance. They tolerated concentrations as high as up to 20 mM As, 2 mM Cd, 6 mM Cu and Zn.

**Table 4:** Maximum tolerable concentration of metal/loid showed by selected strains.

Strain	Cd (mM)	As (mM)	Cu (mM)	Zn (mM)
N4	0.7	1	3	1.9
N8	0.4	2.5	1.9	1.8
N10	0.4	0.4	1.4	2.5
N12	0.1	0	1.8	1.4

N4: *Pseudomonas* sp. N4; N8: *Pseudomonas* sp. N8; N10: *Ensifer* sp. N10; N12: *Ensifer* sp. N12.

#### Selection of endophytes

Among isolated endophytes, only N10 and N12 strains belonging to *Ensifer* genus were rhizobia able to nodulate *M. sativa*, so they were selected for assays *in planta*. N4 and N8 were selected as nodule enhancing bacteria (NEB) candidates based on their PGP properties and enzymatic activities, since they showed all the properties assayed and 2 enzymatic activities. They were also able to tolerate moderate levels of the assayed metal/loids (Table 4).

### *In vitro* effect of endophytes in *Medicago sativa* germination and nodulation

**Figure 1:**
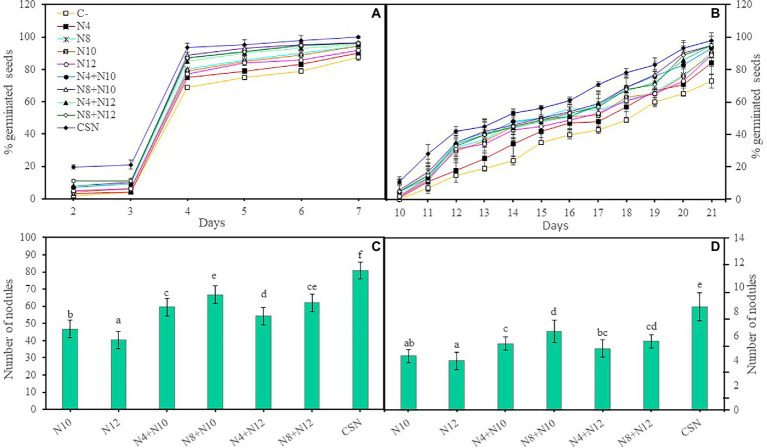
*In vitro* effects of inoculation of *M. sativa* with selected PGPNE. **(A)** Percentage of germinated seeds without metals and **(B)** with metals. Values are means ± S.D. (*n* = 50). **(C)** Number of nodules in plants without As and **(D)** with As. Values are means ± S.D. (*n* = 5). Different letters indicate statistical differences between means (One-way ANOVA, LSD test, *p* < 0.0001). C-: non inoculation; N4: inoculation with *Pseudomonas* sp. N4; N8: inoculation with *Pseudomonas* sp. N8; N10: inoculation with *Ensifer* sp. N10; N12: inoculation with *Ensifer* sp. N12; N4 + N10: co-inoculation with *Pseudomonas* sp. N4 and *Ensifer* sp. N10; N8 + N10: co-inoculation with *Pseudomonas* sp. N8 and *Ensifer* sp. N10; N4 + N12: co-inoculation with *Pseudomonas* sp. N4 and *Ensifer* sp. N12; N8 + N12: co-inoculation with *Pseudomonas* sp. N8 and *Ensifer* sp. N12; CSN: inoculation with *Pseudomonas* sp. N4, *Pseudomonas* sp. N8, *Ensifer* sp. N10 and *Ensifer* sp. N12.

The germination of *M. sativa* seeds was evaluated in the presence and absence of a mixture of metals/loids by inoculating them with the selected strains, performing individual inoculations (N4, N8, N10 and N12), co-inoculations combining each of the *Pseudomonas* and each of the rhizobia (N4 + N10, N8 + N10, N4 + N12, and N8 + N12) or inoculation with the four strains together (N4 + N8 + N10 + N12). Inoculation improved seed germination and the consortium with the four strains reported the greatest increase, showing the highest percentage of germination both in absence and presence of metals (Figures 1A,B). The increase in the germination rate in absence or presence of metals, followed this pattern: CSN > N8 + N10 > N4 + N10 > N8 + N12 > N4 + N12 > N8 > N10 > N12 > N4 > C-. In absence of As, differences in germination among seeds inoculated with the four strains and those co-inoculated con *Pseudomonas* and *Ensifer* were not statistically significant (*p* < 0.001), but these germination rates were significantly higher than those recorded in seeds inoculated with a single strain. Seeds inoculated with single strains did not show significant differences in germination rates among them. The same results could be observed in presence of As, with one single difference, since seeds inoculated with the consortium showed significantly higher germination rates than seeds co-inoculated with *Pseudomonas* and *Ensifer* (p < 0,001). Non-inoculated seeds showed the lowest rates of seed germination, with significant differences, both in absence and presence of As. These significant differences were recorded at the end of the experiment.

To evaluate the ability of the *Pseudomonas* strains to improve *M. sativa* nodulation induced by *Ensifer*, plant seedlings were inoculated or co-inoculated with combinations of *Pseudomonas* and *Ensifer* strains in square plates with or without As. Co-inoculation with either of the *Pseudomonas* and a *Ensifer* strain significantly increased the number of nodules, both in absence and presence of As, compared with the single inoculation with *Ensifer* (Figures 1D,E). The combination *Pseudomonas* sp. N8 and *Ensifer* sp. N10 induced more nodules than any other couple, although the plants inoculated with the combination of the four strains (CSN) showed the highest number of nodules in both conditions, with significant differences. *Ensifer* sp. N10 showed a better behavior in nodulation than N12 and *Pseudomonas* sp. N8 seemed to be better as nodule enhancing bacteria than N4.

### *Pseudomonas* behave as nodule endophytes in *Medicago sativa*

**Figure 2:**
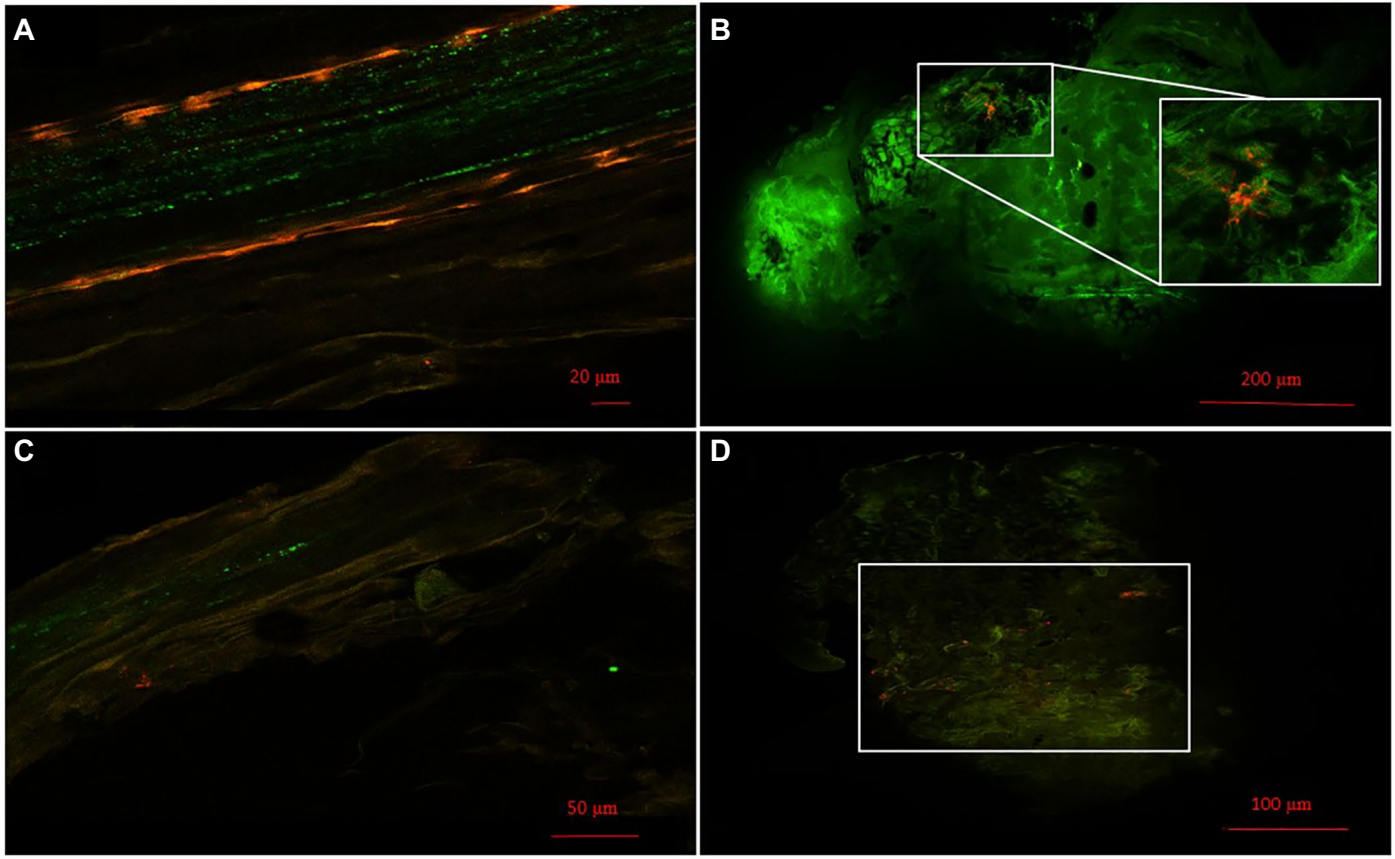
Bacterial colonization. **(A)** Images of roots and **(B)** nodules of colonized *M. sativa* 28 days after inoculation with *Pseudomonas* sp. N4 marked with mCherry and *Ensifer* sp. N10. **(C)** Images of roots and **(D)** nodules of colonized *M. sativa* 28 days after inoculation with *Pseudomonas* sp. N8 marked with mCherry and *Ensifer* sp. N10. The white square in **(B,D)** marks a group of *Pseudomonas* sp. N4 and *Pseudomonas* sp. N8, respectively, in the nitrogen fixation zone.

Localization of *Pseudomonas* strains was studied using confocal laser scanning microscopy (CLSM) and mCherry-labeled *Pseudomonas* sp. N4 and N8. Cells were visualized from 0.5 mm sections of roots and nodules of *M. sativa* co-inoculated with *Ensifer* sp. N10 and labelled N4 or N8. Examples of the results obtained are presented in Figure 2. The presence of labelled bacteria with red fluorescence could be observed both in root (Figures 2A,C) and nodule cells (Figures 2B,D). Groups of bacteria were clearly seen in the nitrogen fixation zone of *M. sativa* nodules, marked with a white square in the figures (Figures 2B,D).

### Effect of inoculations under greenhouse conditions

#### Inoculation increased plant biomass

**Figure 3:**
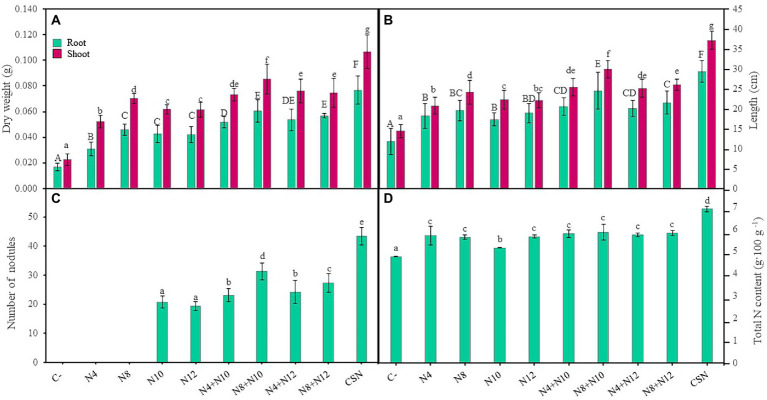
Effects of the inoculation of *M. sativa* plants with PGPNE. **(A)** Dry weight of shoot and roots, **(B)** length of shoot and roots, **(C)** number of nodules, and **(D)** nitrogen content after 60 days in pots containing soil from the high marshes of the Odiel River. Values are means ± S.D. (*n* = 16). Different letters indicate statistical differences between means. Lowercase and uppercase letters are used to qualify different variables and are not comparable among them (One-way ANOVA, LSD test, *p* < 0.001). C-: non inoculation; N4: inoculation with *Pseudomonas* sp. N4; N8: inoculation with *Pseudomonas* sp. N8; N10: inoculation with *Ensifer* sp. N10; N12: inoculation with *Ensifer* sp. N12; N4 + N10: co-inoculation with *Pseudomonas* sp. N4 and *Ensifer* sp. N10; N8 + N10: co-inoculation with *Pseudomonas* sp. N8 and *Ensifer* sp. N10; N4 + N12: co-inoculation with *Pseudomonas* sp. N4 and *Ensifer* sp. N12; N8 + N12: co-inoculation with *Pseudomonas* sp. N8 and *Ensifer* sp. N12; CSN: inoculation with *Pseudomonas* sp. N4, *Pseudomonas* sp. N8, *Ensifer* sp. N10 and *Ensifer* sp. N12.

*M. sativa* plants were grown and inoculated under greenhouse conditions using soil from the marshes of the Odiel River (Huelva). Plant inoculation with any of the strains increased biomass, both shoots and roots, compared with non-inoculated control plants (Figure 3A). Single inoculation with *Pseudomonas* sp. N8 reported higher plant shoot biomass than single inoculations with *Ensifer* or N4, that showed the lowest shoot and root biomasses among single inoculated plants. No significant differences in root biomass were found among plants inoculated with N8, N10 and N12. Co-inoculation with *Pseudomonas-Ensifer* couples produced higher values of root and shoot biomasses than any of the single inoculation treatments, except for plants inoculated with N4-N10 couple, that did not show significant differences in shoot biomass compared with plants inoculated with N8. N8 + N10 combination showed the highest value in plant shoot biomass among the *Pseudomonas-Ensifer* co-inoculation treatments, with significant differences (Figure 3A). Nevertheless, the highest values of root and shoot biomass were recorded in plants inoculated with the consortium of the four strains (CSN). Similitudes could be observed using plant root and shoot lengths as parameters to compare inoculation treatments (Figure 3B). Plants co-inoculated with *Pseudomonas* sp. N8 and *Ensifer* reported longer shoots than those inoculated with a single strain, while the combination N8 + N10 showed the longest roots and shoots among plants co-inoculated or inoculated with a single strain. Those inoculations including *Ensifer* and N4 showed no significant differences in root and shoot length compared with plants inoculated only with N8. The longest roots and shoots were observed in plants inoculated with the consortium (Figure 3B). These plants also presented the highest number of leaves with the widest diameter, with statistically significant differences compared to non-inoculated controls or any of the inoculation and co-inoculation treatments (Supplementary Figure 2).

#### *Pseudomonas* enhanced plant nodulation

Regarding the number of nodules, *Pseudomonas* increased the number of nodules induced by *Ensifer*, showing co-inoculated plants more nodules than plants inoculated only with *Ensifer*, with significant differences (Figure 3C). Although this result was independent of the *Ensifer*-*Pseudomonas* combination used, plants co-inoculated with strains N8 and N10 showed higher number of nodules than any other couple. The highest number of nodules was found again in plants inoculated with the consortium (Figure 3C), approximately 109 and 124% more nodules than plants inoculated with N10 and N12, respectively. Nitrogen content in stems and leaves of *M. sativa* plants was also evaluated (Figure 3D). Inoculation treatments increased the N content compared to non-inoculated plants, although no significant differences were found among single inoculation and co-inoculation treatments, except for single inoculation with N10, that reported lower values of N (Figure 3D). The highest content of N, with significant differences, was measured in plants inoculated with the consortium of the four strains.

#### *Pseudomonas* ameliorated the physiological state of the plants

**Figure 4:**
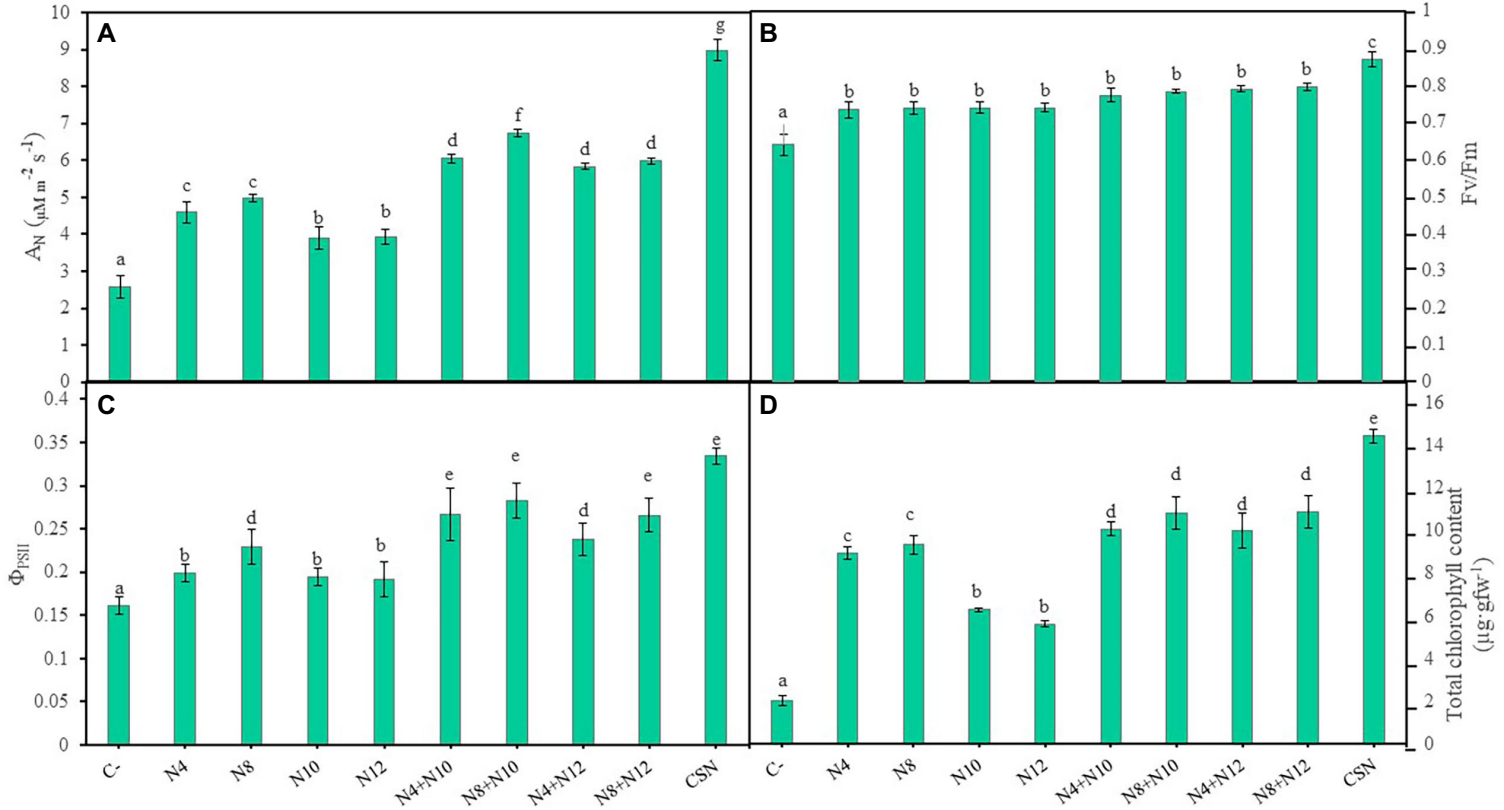
Photosynthetic parameters of *M. sativa* plants inoculated with PGPNE. **(A)** Net photosynthetic rate (A_N_), **(B)** maximum quantum efficiency of PSII photochemistry (Fv/Fm), **(C)** quantum yield of PSII photochemistry (Φ_PSII_), and **(D)** total chlorophyll content after 60 days in pots containing soil from the high marshes of the Odiel River. Values are means ± S.D. (*n* = 16). Different letters indicate statistical differences between means (One-way ANOVA, LSD test, *p* < 0.0001). C-: non inoculation; N4: inoculation with *Pseudomonas* sp. N4; N8: inoculation with *Pseudomonas* sp. N8; N10: inoculation with *Ensifer* sp. N10; N12: inoculation with *Ensifer* sp. N12; N4 + N10: co-inoculation with *Pseudomonas* sp. N4 and *Ensifer* sp. N10; N8 + N10: co-inoculation with *Pseudomonas* sp. N8 and *Ensifer* sp. N10; N4 + N12: co-inoculation with *Pseudomonas* sp. N4 and *Ensifer* sp. N12; N8 + N12: co-inoculation with *Pseudomonas* sp. N8 and *Ensifer* sp. N12; CSN: inoculation with *Pseudomonas* sp. N4, *Pseudomonas* sp. N8, *Ensifer* sp. N10 and *Ensifer* sp. N12.

Several photosynthetic parameters were measured in order to determine the physiological state of the plants (Figure 4; Supplementary Figure 3). Inoculated plants showed higher values in all the parameters recorded compared to non-inoculated plants and the highest values were always measured in plants inoculated with the consortium, with significant differences. Values in the net photosynthetic rate (A_N_) could be described by the following pattern, with significant differences: CSN > N8 + N10 > N8 + N12 = N4 + N10 = N4 + N12 > N8 = N4 > N12 = N10 > C- (Figure 4A). With the exception of the inoculation with the consortium, no differences were found in the maximum quantum efficiency of the PSII photochemistry (Fv/Fm) among inoculation conditions (Figure 4B). Concerning the quantum yield of the PSII photochemistry (Φ_PSII_), results followed the pattern: CSN > N8 + N12 = N8 + N10 = N4 + N10 > N4 + N12 = N8 > N12 = N10 = N4 > C- (Figure 4C). In the same way, plants inoculated with the consortium showed the highest values in electron transport rate (ETR), followed by plants co-inoculated with N8 + N10 (Supplementary Figure 3). Finally, values of total chlorophyll content in plants could be described by a pattern similar to the one observed for Fv/Fm: CSN > N8 + N12 = N4+ N12 = N8 + N10 = N4 + N10 > N8 = N4 > N12 = N10 > C- (Figure 4D).

#### *Pseudomonas* increased the activity of plant antioxidant enzymes

**Figure 5:**
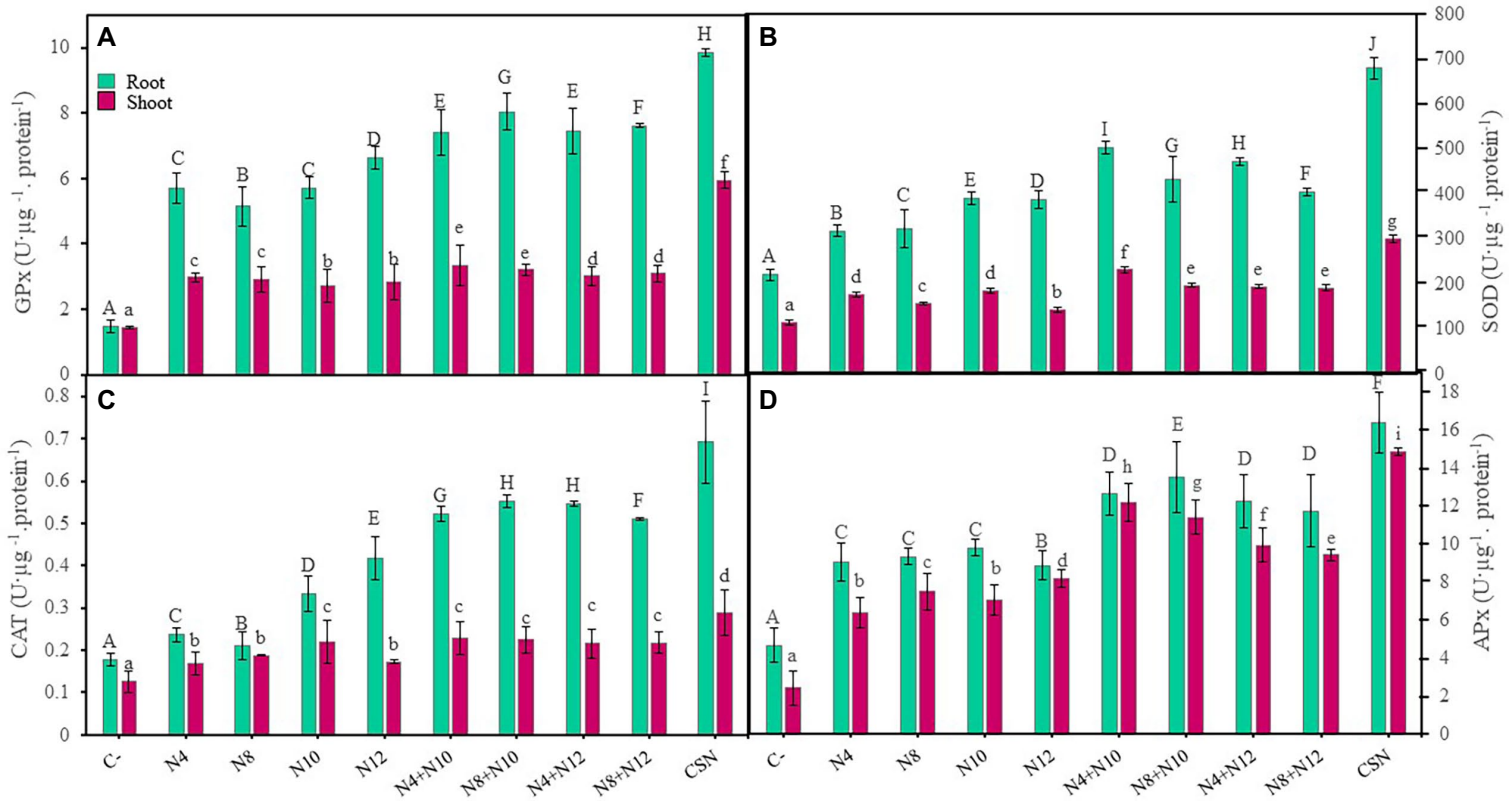
Antioxidant enzymes in *M. sativa* plants inoculated with PGPNE. **(A)** Guaiacol peroxidase, **(B)** superoxide dismutase, **(C)** catalase, and **(D)** ascorbate peroxidase activities after 60 days in pots containing soil from the high marshes of the Odiel River. Values are means ± S.D. (*n* = 16). Different letters indicate statistical differences between means. Lowercase and uppercase letters are used to qualify different variables and are not comparable among them (One-way ANOVA; LSD test, *p* < 0.001). C-: non inoculation; N4: inoculation with *Pseudomonas* sp. N4; N8: inoculation with *Pseudomonas* sp. N8; N10: inoculation with *Ensifer* sp. N10; N12: inoculation with *Ensifer* sp. N12; N4 + N10: co-inoculation with *Pseudomonas* sp. N4 and *Ensifer* sp. N10; N8 + N10: co-inoculation with *Pseudomonas* sp. N8 and *Ensifer* sp. N10; N4 + N12: co-inoculation with *Pseudomonas* sp. N4 and *Ensifer* sp. N12; N8 + N12: co-inoculation with *Pseudomonas* sp. N8 and *Ensifer* sp. N12; CSN: inoculation with *Pseudomonas* sp. N4, *Pseudomonas* sp. N8, *Ensifer* sp. N10 and *Ensifer* sp. N12.

The influence of bacterial inoculations on plant response to stress was evaluated by determining the activity of different antioxidant enzymes in roots and leaves of *M. sativa* (Figure 5). All the enzymatic activities increased in response to inoculation, both in roots and shoots, compared to non-inoculated plants, with higher increases in roots than in shoots. The highest enzymatic activities were always recorded in plants inoculated with the consortium, with significant differences. Guaiacol peroxidase (Figure 5A) and ascorbate peroxidase (Figure 5D) activities presented similar results, showing plants inoculated with N8 + N10 the highest levels of activity in roots among plants co-inoculated or inoculated with single strains. Concerning catalase activity, plants co-inoculated with *Pseudomonas-Ensifer* couples had higher levels of activities in roots and shoots than those inoculated only with one strain, with the single exception of plants inoculated with N10, that showed the same level activity in shoots than co-inoculated plants (Figure 5C). Finally, plants inoculated with the combination N4 + N10 showed more superoxide dismutase activity in roots and shoots than any other inoculation condition, with the exception of plants inoculated with the consortium (Figure 5B).

#### *Pseudomonas* increased metal accumulation in roots

**Figure 6:**
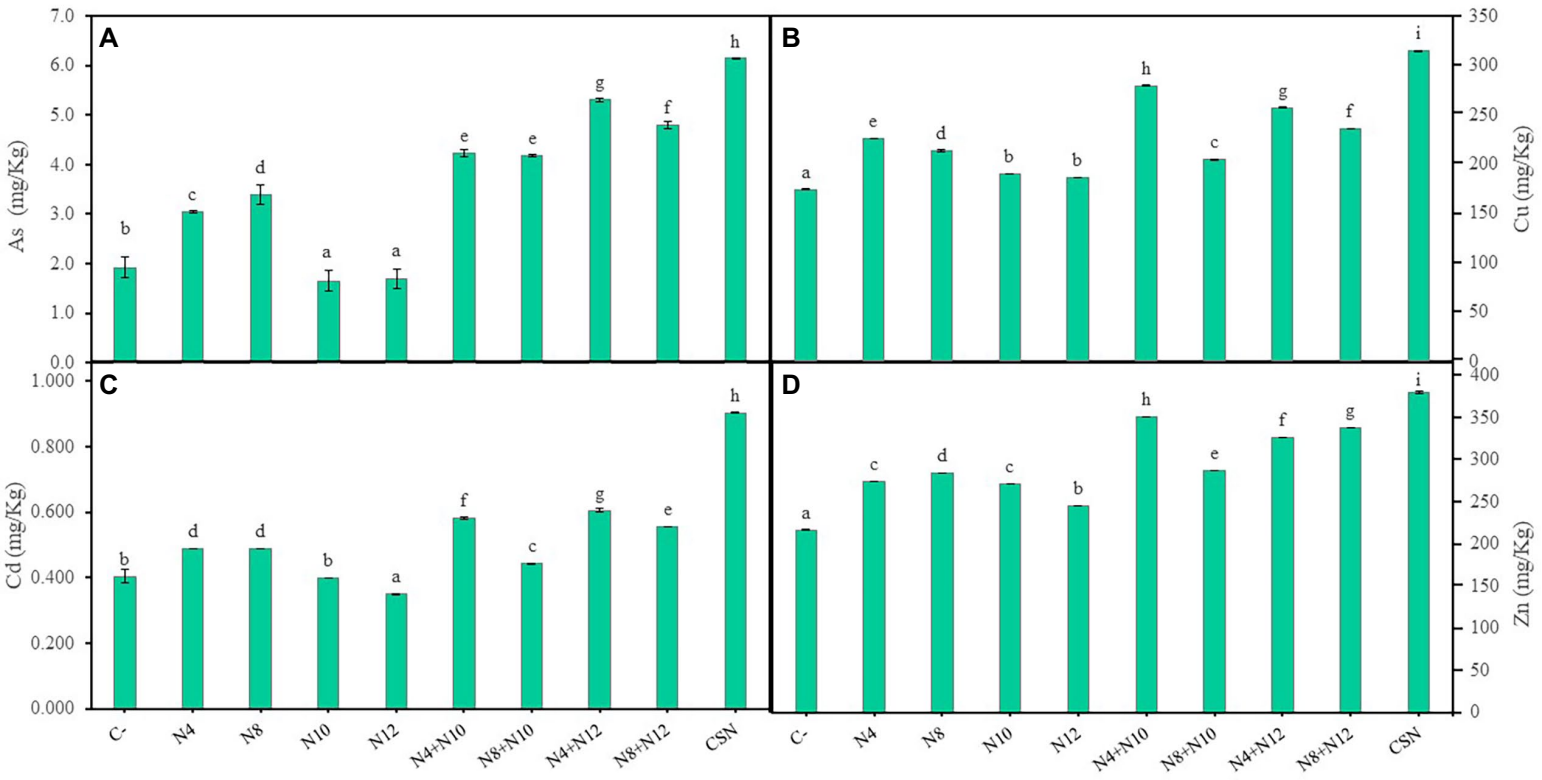
Accumulation of metal/loids in roots of *M. sativa*. **(A)** Accumulation of arsenic, **(B)** copper, **(C)** cadmium, and **(D)** zinc after 60 days in pots containing soil from the high marshes of the Odiel River. Values are means ± S.D. (*n* = 16). Different letters indicate statistical differences between means (One-way ANOVA; LSD test, *p* < 0.001). C-: non inoculation; N4: inoculation with *Pseudomonas* sp. N4; N8: inoculation with *Pseudomonas* sp. N8; N10: inoculation with *Ensifer* sp. N10; N12: inoculation with *Ensifer* sp. N12; N4 + N10: co-inoculation with *Pseudomonas* sp. N4 and *Ensifer* sp. N10; N8 + N10: co-inoculation with *Pseudomonas* sp. N8 and *Ensifer* sp. N10; N4 + N12: co-inoculation with *Pseudomonas* sp. N4 and *Ensifer* sp. N12; N8 + N12: co-inoculation with *Pseudomonas* sp. N8 and *Ensifer* sp. N12; CSN: inoculation with *Pseudomonas* sp. N4, *Pseudomonas* sp. N8, *Ensifer* sp. N10 and *Ensifer* sp. N12.

Accumulation of As and the most abundant metals in the high marshes of the Odiel river was determined in *M. sativa* tissues at the end of the greenhouse experiment (Figure 6). The highest levels of metal/loids found in shoots were 1.5 ppm of As, 0.04 ppm of Cd, 27.76 ppm of Cu and 61.2 ppm of Zn, without significant differences among inoculation conditions (Supplementary Table 6). Different behavior was observed in roots. Single inoculations with rhizobia (N10 and N12) reduced As accumulation in roots compared to control plants (Figure 6A). Plants inoculated with rhizobia also showed the lowest values of Cu and Cd accumulated in roots compared with the rest of inoculation conditions (Figures 6B,C). Concerning Zn, the lowest amounts were recorded in plants inoculated with N12 and the highest in plants inoculated with N8, while plants inoculated with the other strains (N4 and N10) accumulated the same amount of this metal (Figure 6D). On the contrary, the highest values of metal accumulation were found in roots of plants inoculated with the consortium of the four bacteria (Figure 6). In general, plants inoculated with *Pseudomonas*-*Ensifer* couples accumulated more As and metals in roots than those inoculated only with *Pseudomonas*, with the exceptions of Cu and Cd in plants inoculated with the couple N8-N10 (Figure 6).

## Discussion

Climate change and human activities are causing abiotic stress in many soils previously suitable for crops. Use of agrochemicals, heavy metals release, drought or salinity cause a great loss of nutrients and native microorganisms in the soil, altering plant adaptation and growth (García-Martí et al., 2019; Du et al., 2021). Environmentally friendly solutions to regenerate soils should include the use of leguminous plants, since they are high biomass producers, reduce the use of nitrogen-based fertilizers and can be used for fed or feeder in agriculture (Graham and Vance, 2003; Ferreira et al., 2021). In addition, most legumes are metal phytostabilizers suitable for regeneration of metal contaminated soils (Jach et al., 2022). Legume adaptation and growth in nutrient-poor or degraded soils can be enhanced using adequate autochthonous rhizospheric or endophytic PGPB as inoculants (Monteoliva et al., 2022).

The objective pursued in this work was the isolation and characterization of endophytic bacteria from nodules of *Medicago* spp. plants growing in the estuary of the Odiel river (Huelva, Spain), capable of improving seed germination, nodulation and growth of *M. sativa* in soils with low nutrient content and moderate to high levels of heavy metals. 33 strains belonging to genera previously described as nodule endophytes, such as *Achromobacter, Bacillus, Enterobacter, Lelliottia, Pantoea, Priestia* or *Pseudomonas* (Aserse et al., 2013; Martínez-Hidalgo and Hirsch, 2017), were isolated. Among NRE, *Pseudomonas* was the most represented genus and only two isolated were nodule inducing rhizobia from *Ensifer* genus, the most common *Medicago* symbiont frequently isolated from wild legumes growing in arid soils (Mnasri et al., 2007; León-Barrios et al., 2009; Bessadok et al., 2020).

Characterization of the isolates revealed the presence of at least 4 out of 6 PGP properties studied, several enzymatic activities and moderate to high levels of As and metals tolerance in all of them. Strains *Pseudomonas* sp. N4 and *Pseudomonas* sp. N8, showing all the PGP properties assayed, were selected for *in planta* experiments to determine their potential as nodule enhancing endophytes (NEE) in co-inoculation with *Ensifer* sp. N10 and *Ensifer* sp. N12. In this work, strains N4 and N8 have proved to be nodule endophytes, since fluorescent m-Cherry labelled strains were observed inside *M. sativa* roots and nodules induced by *Ensifer*.

Inoculation with the selected endophytes improved *M. sativa* seeds germination both in presence and absence of As and metals. Several properties may have contributed to this effect: (i) ACC deaminase activity, observed in N4 and N8, that regulates stress level in plants through ethylene degradation (Penrose and Glick, 2003; Chandwani and Amaresan, 2022), (ii) IAA produced by the selected strains, a hormone involved in plant development (Khan et al., 2020; Hu et al., 2021), and (iii) cellulase activity presents in strains N8, N10 and N12, a lytic enzyme that could participate in plant cell wall degradation during seed germination (Flores-Duarte et al., 2022b). The combined effect of these and other properties would also explain why co-inoculation experiments showed better results than single inoculation with one strain and the highest percentage of germination was observed in seeds inoculated with the consortium of the four strains.

The ability of the selected NEE to promote *M. sativa* growth and nodulation in greenhouse conditions was evaluated in soils collected from Odiel river marshes, with low levels of nutrients and moderate to high amounts of As and metals. Inoculation with any of the strains improved plant biomass, root and shoot length and number and diameter of the leaves. This improvement was generally higher in plants co-inoculated with *Pseudomonas*-*Ensifer* couples than in plants inoculated with one single strain. The highest plant weights and heights were recorded in plants inoculated with the four strains. The increase in the length of plant roots and shoots may be related to the production of IAA (Khan et al., 2020), that plays an essential role in the development and growth of plants, by increasing cell expansion and is an essential hormone in root development (Hu et al., 2021). Endophyte properties related with nutrient acquisition could also be involved in these results. P availability in the soil is a limiting factor for plant growth and development (Timofeeva et al., 2022). Endophytes could provide phosphorus through phosphate solubilization (Mei et al., 2021; Zhang et al., 2022). In legumes, P helps in the nodulation process, amino acid and proteins synthesis (Wang et al., 2020). Through siderophore production endophytes can provide essential metals such iron and Zn (Mahanty et al., 2017). Other related properties that influence legume growth could be biofilm formation, that is an important mechanism during bacterial attachment to the root, facilitating root colonization and endophyte entry and, at the same time, enhancing nutrient absorption (Das et al., 2012). Finally, the production of lytic enzymes by endophytes could benefit plant growth. These enzymes are essential in the symbiosis between plants and microorganisms, since they contribute to the degradation of organic matter, the colonization of bacteria, the acquisition of nutrients (Wang and Dai, 2010; Eid et al., 2021), and the degradation of starch as an energy source in the germination stage (Walitang et al., 2017).

Concerning nitrogen, co-inoculation with *Ensifer* and *Pseudomonas* increased the number of nodules induced by *Ensifer* in single inoculations, both in presence and absence of metals, *in vitro* and in greenhouse conditions. Nevertheless, there were no significant differences in the amount of nitrogen accumulated in shoots among inoculation conditions, except in plants inoculated with the consortium that showed the highest levels of nitrogen with significant differences. ACC deaminase activity present in *Pseudomonas* could have facilitated the reduction of the stress levels in the the plants by reducing ethylene (Singh et al., 2022), which in excess causes defoliation, senescence and inhibit cell elongation, among other effects (Fatma et al., 2022). In that way, ACC deaminase favors germination, nodulation, and the development of plants (Singh et al., 2022). ACC deaminase activity and IAA production also play an interesting role in the nodulation process by delaying the senescence of the nodule, creating an interaction with the bacteroid (Alemneh et al., 2020). Strains N4 and N8 grew in a nitrogen-free medium, suggesting that they could fix atmospheric nitrogen. In a work in progress, nitrogenase genes (*nifKDH*) have been amplified in these strains (data not shown). This could explain the high levels of nitrogen measured in plant inoculated with *Pseudomonas*.

The positive effects of *Pseudomonas-Ensifer* co-inoculation on plant development were also reflected in the physiological state of the plants, since co-inoculations improved most of the photosynthetic parameters analyzed: A_N_, Φ_PSII_, ETR and total chlorophyll, except for plants co-inoculated with N4 + N12 plants, that showed the same values of Φ_PSII_ than plants inoculated with N8. In addition, plants inoculated with the consortium of the four strains showed the highest efficiency of photosystem II (Φ_PSII_), the best balance of carbon and water assimilation, the greatest efficiency in the use of energy from the photochemical apparatus, and the biggest increase in total chlorophyll (Liu et al., 2020).

In this work, the levels of stress in *M. sativa* plants were measured by recording the activity of antioxidant defense enzymes, particularly catalase, guaiacol peroxidase, ascorbate peroxidase and superoxide dismutase, that act as protectors against reactive oxygen species (ROS). An excess of ROS production as well as a decrease in the antioxidant defense mechanism causes a breakdown of cell function, damaging the plant and reducing its development (Redondo-Gómez et al., 2011; Mesnoua et al., 2016). Our results showed higher enzymatic activities in plants co-inoculated with *Ensifer* and *Pseudomonas* than in single inoculated plants and the highest activities in plants inoculated with the consortium of the four bacteria. This pointed to the role of these bacteria in regulating stress mechanisms in plants. Induction of antioxidant enzymatic activities after inoculation with rhizospheric bacteria has been reported in plants of *M. sativa* under heavy metal stress (Raklami et al., 2019) and nutrient deficiency (Flores-Duarte et al., 2022b), and in crops such as maize, where inoculated plants showed an increase of catalase, peroxidase, and superoxide dismutase activities (Kukreti et al., 2020; Chaudhary et al., 2021; Agri et al., 2022). Concerning endophytes, increased enzymatic activities have been reported recently in *M. sativa* plants growing under stress conditions and inoculated with a bacterial consortium containing *Variovorax* nodule endophytes (Flores-Duarte et al., 2022a).

The use of legumes and associated rhizobia is an interesting tool to fight against soil metal contamination, since legume interacting with rhizobia have the ability to accumulate high concentrations of metals particularly in roots, with low levels of translocation to shoots, without disturbing plant growth (Jach et al., 2022). In this work, the highest levels of As, Cd, Cu and Zn measured in shoots of *M. sativa* were much below those allowed for human or animal consumption (Junta de Andalucía and Consejeria de Medio Ambiente, 1999), indicating that our strategy would not be dangerous for living beings in terms of metals mobilization. Regarding the accumulation of metals/loids in the roots, inoculation with *Pseudomonas* or *Pseudomonas-Ensifer* couples promoted metal accumulation and the highest levels of As, Cd, Cu and Zn were measured in *M. sativa* roots inoculated with the consortium of the four strains (CSN). In that way, inoculation with *Pseudomonas* strains enhanced the metal phytostabilization potential of *M. sativa* plants in soils with moderate to high levels of metals, without negative effects on plant growth.

Although combinations of *Pseudomonas* and *Ensifer* strains, and particularly those including N8, had a significant positive effect on *M. sativa* growth and nodulation, the best results were always recorded in plants inoculated with the consortium containing the four strains, demonstrating the advantages of using a consortium with several strains and the collaborative effects of their properties.

A consortium of three rhizospheric bacteria able to promote *M. sativa* growth and adaptation in estuarine soils with nutrients poverty has been recently reported (Flores-Duarte et al., 2022b). These PGPB inoculated as consortium in poor-nutrient soils increased plant shoot biomass (around 100%) and ameliorated *M. sativa* nodulation (around 50%) compared to plants inoculated only with rhizobia. Although we have to consider differences between soils to establish comparisons, particularly due to levels of metal contamination, in the current work our consortium of endophytes increased plant shoot biomass around 100% and also ameliorated *M. sativa* nodulation around 100% compared to plants inoculated only with the best performing rhizobia for each parameter. Despite these differences in nodulation improvement, both consortiums induced similar increases in plant N content (around 1 g/100 g). Inoculation of *M. sativa* with the rhizospheric consortium also induced positive effects on plant photosynthetic status under nutrient deficiency (Flores-Duarte et al., 2022b). All the photosynthetic parameters recorded showed increased values in plants inoculated with the consortium compared to plants inoculated only with rhizobia, even though, parameters such as A_N_ or Φ_PSII_ showed no significant differences (Flores-Duarte et al., 2022b). In this work, significant differences in the levels of all the photosynthetic parameters recorded, including A_N_ and Φ_PSII_, were observed between plants inoculated with the consortium and those inoculated with rhizobia, suggesting that endophytes were more efficient in enhancing the photosynthetic status of the plant than rhizospheric bacteria.

The presence of moderate to high levels of metals in the soil, increasing *M. sativa* stress conditions, required the isolation and characterization of specific bacteria able to help the plant to deal with these particular environmental conditions. Our results suggest that inoculants based on non-rhizobial nodule endophytes could be useful and efficient tools to enhance legume adaptation and growth in metal contaminated and nutrient-poor soils. At least under stress conditions, endophytes could provide some advantages as PGPB compared to rhizospheric bacteria, in addition to their narrower relationship with the plant and the lack of competence with soil bacteria (Adeleke and Babalola, 2021; Dwibedi et al., 2022). In that way, it looks necessary to investigate microbiomes of a wide diversity of nitrogen-fixing nodules to find useful inoculants to be applied in different environmental conditions (Martínez-Hidalgo and Hirsch, 2017).

## Conclusion

Autochthonous *Pseudomonas* sp. N4 and *Pseudomonas* sp. N8 strains enhanced growth, nodulation and metal accumulation in roots of *M. sativa* plants inoculated with wild type *Ensifer* strains in soils with low nutrients content and moderate to high levels of metals, ameliorating the physiological state of the plants and helping to regulate plant stress mechanisms, thus facilitating plant adaptation to these abiotic stresses. Our results suggest that selected native PGPNE (plant growth promoting nodule endophytes) could be adequate biotools to promote legume adaptation, growth and phytostabilization potential in nutrient-poor and/or metal contaminated estuarine soils.

## Data availability statement

The datasets presented in this study can be found in online repositories. The names of the repository/repositories and accession number(s) can be found at: https://www.ncbi.nlm.nih.gov/, OP060607-OP0606030.

## Author contributions

IR-L and SN-T: conceptualization and writing—original draft preparation. NF-D and SC-D: methodology and investigation. SN-T: software. SN-T and NF-D: formal analysis. EM-N and SR-G: data curation. IR-L, EM-N, and EP: writing—review and editing and funding acquisition. IR-L and SR-G: supervision. EP: project administration. All authors contributed to the article and approved the submitted version.

## Funding

This research was funded by MCIN, project MCIN/AEI/10.13039/501100011033, UE “NextGenerationEU/PRTR (PDC2021-120951-I00) and Junta de Andalucía, I + D + I FEDER Andalucía project US-1262036 and PAIDI2020, project P20_00682.

## Conflict of interest

The authors declare that the research was conducted in the absence of any commercial or financial relationships that could be construed as a potential conflict of interest.

## Publisher’s note

All claims expressed in this article are solely those of the authors and do not necessarily represent those of their affiliated organizations, or those of the publisher, the editors and the reviewers. Any product that may be evaluated in this article, or claim that may be made by its manufacturer, is not guaranteed or endorsed by the publisher.
