# Supplementary material for: Enhanced legume growth and adaptation to degraded estuarine soils using Pseudomonas sp. nodule endophytes
Source: Front Microbiol. 2022 Oct 20;13:1005458. doi: 10.3389/fmicb.2022.1005458 (PMC9631207; doi:10.3389/fmicb.2022.1005458)
Supplement: Supplementary file 1 [file Data_Sheet_1.docx]

Supplementary Material

| **Supplementary Table 1.** Strains used in PGP properties and enzymatic activities tests as controls. | | | | |
| --- | --- | --- | --- | --- |
| **Strain** | **Accession number** | **Type of control** | **Activity** | **Reference** |
| SMJ25 | KT036405 | Positive | All enzymatic activities, except amylase and pectinase | Mesa et al., 2015a |
| SMJ30 | KT036407 | Positive | Siderophores production, phosphate solubilization, and nitrogen fixation |  |
| SMT38 | KF962966 | Positive | Biofilm formation and ACC deaminase | Mesa et al., 2015b |
| SMT46 | KF962974 | Negative | All PGP properties |  |
| RA15 | KU588400 | Positive | Pectinase | Navarro-Torre et al., 2016 |
| EAod10 | KU320863 | Positive | Amylase activity and IAA production |  |
| RA18 | KU588386 | Negative | All enzymatic activities |  |

| **Supplementary Table 2**. Morphological characteristics of the endophytes isolated from nodules of *Medicago* spp. Selected strains appear in bold. | |
| --- | --- |
| **Rod-shape and short Gram- negative** | N1, N2, N3, **N4**, N5, N6, N7, **N8**, N9, **N10**, N11, **N12**, N13, N15, N16, N17, N19, N20, N21, N22, N23, N24, N26, N28, N29, N30, N31, N33 |
| **Rod-shape and short Gram- positive** | N14, N18, N25, N32 |
| **Rod-shape and short sporulated Gram- positive** | N27 |

| **Supplementary Table 3**. PGP properties showed by the endophytes isolated from nodules of *Medicago* spp. Selected strains appear in bold. | | | | | | |
| --- | --- | --- | --- | --- | --- | --- |
| **Strain** | **Phosphate solubilisation** | **Siderophore production** | **IAA production** | **Biofilm** | **ACC deaminase activity** | **Nitrogen fixation** |
| N1 | - | 13 | - | + | 3.645 | + |
| N2 | 12 | 35 | 8.974 | + | - | + |
| N3 | 10 | 50 | 7.789 | + | - | + |
| **N4** | **11** | **73** | **1.562** | **+** | **9.677** | **+** |
| N5 | 12 | 40 | 3.425 | + | - | + |
| N6 | - | 15 | - | + | - | + |
| N7 | - | 25 | 2.506 | + | - | + |
| **N8** | **12** | **44** | **17.939** | **+** | **9.987** | **+** |
| N9 | 12 | 39 | 5.995 | + | - | + |
| **N10** | **15** | **13** | **1.266** | **+** | **-** | **-** |
| N11 | 17 | 20 | 16.158 | + | - | + |
| **N12** | **14** | **10.2** | **1.001** | **+** | **-** | **-** |
| N13 | 11 | 42 | 15.459 | + | - | + |
| N14 | 15 | 18 | 16.884 | + | - | + |
| N15 | 21 | 30 | - | - | - | + |
| N16 | 15 | 35 | - | - | 5.642 | - |
| N17 | 18 | 45 | - | - | 4.266 | - |
| N18 | 17 | 33 | 2.326 | + | - | + |
| N19 | 13 | 50 | 6.596 | - | 5.576 | + |
| N20 | 23 | 15 | 7.287 | + | - | + |
| N21 | 17 | 60 | - | - | - | + |
| N22 | 13 | 45 | 8.121 | - | - | + |
| N23 | 12 | 15.17 | 7.914 | - | - | + |
| N24 | 12 | 40 | 7.103 | + | - | + |
| N25 | 15 | 22 | 2.592 | + | - | + |
| N26 | 14 | 38 | 7.987 | + | - | + |
| N27 | 12 | 21 | 4.055 | + | - | + |
| N28 | 14 | 20 | 11.931 | - | 2.169 | + |
| N29 | 18 | 21 | 8.905 | - | 6.472 | + |
| N30 | 20 | 45 | 1.484 | - | 4.869 | + |
| N31 | 22 | 50 | 1.639 | - | 1.804 | + |
| N32 | 11 | 24 | 5.296 | - | - | + |
| N33 | 18 | 51 | 3.21 | - | 6.157 | - |
| +, presence of the activity; -, absence of the activity. Values of phosphate solubilisation and siderophores production express the diameter of the halo in mm. Values of IAA production are expressed in mg·L^−1^ . Values of ACC deaminase activity are expressed in µmoles α-ketobutyrate·mg protein^−1^·h^−1^. | | | | | | |

| **Supplementary Table 4**. Enzymatic activities showed by the endophytes isolated from nodules of *Medicago* spp. Selected strains appear in bold. | | | | | | | |
| --- | --- | --- | --- | --- | --- | --- | --- |
| **Strain** | **DNAse** | **Amylase** | **Cellulase** | **Lipase** | **Pectinase** | **Protease** | **Chitinase** |
| N1 | - | - | - | - | - | - | - |
| N2 | - | - | + | - | + | - | - |
| N3 | - | - | + | - | - | - | + |
| **N4** | **-** | **-** | **-** | **-** | **-** | **+** | **-** |
| N5 | - | - | + | - | - | - | - |
| N6 | - | - | - | - | - | - | - |
| N7 | - | - | + | - | - | + | - |
| **N8** | **-** | **-** | **+** | **-** | **-** | **+** | **-** |
| N9 | - | - | + | - | - | - | - |
| **N10** | **+** | **-** | **+** | **-** | **-** | **-** | **-** |
| N11 | - | - | - | - | - | + | - |
| **N12** | **+** | **-** | **+** | **-** | **-** | **-** | **-** |
| N13 | - | - | - | - | - | + | - |
| N14 | - | - | - | - | - | + | - |
| N15 | - | - | - | - | - | + | - |
| N16 | - | - | - | - | - | + | - |
| N17 | - | - | - | - | - | + | - |
| N18 | - | - | + | - | + | + | - |
| N19 | - | - | + | + | - | - | - |
| N20 | - | - | + | + | - | - | - |
| N21 | - | - | + | - | - | + | - |
| N22 | - | - | + | - | + | - | - |
| N23 | - | - | + | - | - | - | - |
| N24 | - | - | + | - | - | - | - |
| N25 | - | - | + | - | - | + | + |
| N26 | - | - | + | - | - | - | - |
| N27 | - | - | + | - | + | + | - |
| N28 | - | - | + | - | - | - | - |
| N29 | - | - | + | - | - | - | - |
| N30 | - | - | - | - | - | + | - |
| N31 | - | - | + | - | - | + | - |
| N32 | - | - | + | - | + | + | - |
| N33 | - | - | - | + | - | + | - |
| +, presence of the activity; -, absence of the activity. | | | | | | | |

| **Supplementary Table 5.** Maximum tolerable concentration of metal/loids showed by the he endophytes isolated from nodules of *Medicago* spp. Selected strains appear in bold. | | | | |
| --- | --- | --- | --- | --- |
| **Strain** | **Cd (mM)** | **As (mM)** | **Cu (mM)** | **Zn (mM)** |
| N1 | 1.8 | 2.9 | 3.4 | 3.9 |
| N2 | 0.6 | 8 | 3.8 | 6 |
| N3 | 0.6 | 8 | 4.4 | 6 |
| **N4** | **0.7** | **1** | **3** | **1.9** |
| N5 | 0.9 | 2.9 | 3.7 | 6 |
| N6 | 1.9 | 17.4 | 3.9 | 3.9 |
| N7 | 1.4 | 8 | 4.4 | 5.2 |
| **N8** | **0.4** | **2.5** | **1.9** | **1.8** |
| N9 | 0.9 | 8 | 1.9 | 5.2 |
| **N10** | **0.4** | **0.4** | **1.4** | **2.5** |
| N11 | 0.2 | 2.9 | 1.9 | 1.9 |
| **N12** | **0.1** | **0** | **1.8** | **1.4** |
| N13 | 0.9 | 8 | 3.6 | 3.9 |
| N14 | 0.1 | 7 | 2 | 1 |
| N15 | 0.9 | 5.8 | 5.3 | 5 |
| N16 | 0.9 | 5.8 | 5.5 | 4 |
| N17 | 1.5 | 5.5 | 5.5 | 5 |
| N18 | 0.125 | 0.4 | 3 | 0.4 |
| N19 | 2 | 20 | 5.5 | 5.8 |
| N20 | 0.4 | 1.8 | 6 | 3 |
| N21 | 0.9 | 8.5 | 5.5 | 5.5 |
| N22 | 1 | 20 | 5.5 | 5.4 |
| N23 | 0.9 | 11 | 5.4 | 2 |
| N24 | 0.6 | 7 | 3.1 | 3 |
| N25 | 2 | 2.5 | 4.1 | 4.5 |
| N26 | 1.9 | 6 | 5.8 | 4.8 |
| N27 | 0.9 | 0.4 | 2 | 2 |
| N28 | 0.5 | 20 | 6 | 2 |
| N29 | 0.9 | 20 | 5.5 | 3 |
| N30 | 0.9 | 7 | 5.8 | 4.5 |
| N31 | 1 | 6 | 5.5 | 5.3 |
| N32 | 0.125 | 3 | 3.1 | 2.1 |
| N33 | 0.9 | 6 | 5.5 | 5.5 |

| **Supplementary Table 6.** Accumulation of metals in shoots of *Medicago sativa*. | | | | |
| --- | --- | --- | --- | --- |
| **Strain** | **As (mg·kg^−1^)** | **Cd (mg·kg^−1^)** | **Cu (mg·kg^−1^)** | **Zn (mg·kg^−1^)** |
| C- | 0.36 ± 0.07^a^ | 0.01 ± 0.06^a^ | 14.86 ± 0.06^a^ | 30.61 ± 0.01^a^ |
| N4 | 1.45 ± 0.02^b^ | 0.03 ± 0.01^b^ | 24.26 ± 0.69^b^ | 50.02 ± 0.56^b^ |
| N8 | 1.49 ± 0.01^b^ | 0.02 ± 0.01^b^ | 25.18 ± 0.54^b^ | 54.03 ± 0.58^b^ |
| N10 | 1.39 ± 0.02^b^ | 0.02 ± 0.00^b^ | 24.80 ± 0.04^b^ | 53.43 ± 0.28^b^ |
| N12 | 1.38 ± 0.01^b^ | 0.03 ± 0.01^b^ | 25.36 ± 0.81^b^ | 52.10 ± 0.68^b^ |
| N4+N10 | 1.44 ± 0.04^b^ | 0.03 ± 0.01^b^ | 22.11 ± 0.21^b^ | 51.13 ± 0.30^b^ |
| N8+N10 | 1.49 ± 0.01^b^ | 0.03 ± 0.00^b^ | 26.21 ± 0.35^b^ | 56.40 ± 0.67^b^ |
| N4+N12 | 1.46 ± 0.00^b^ | 0.03 ± 0.02^b^ | 25.16 ± 0.55^b^ | 55.19 ± 0.81^b^ |
| N8+N12 | 1.43 ± 0.01^b^ | 0.03 ± 0.01^b^ | 23.81 ± 0.05^b^ | 50.43 ± 0.28^b^ |
| CSN | 1.50 ± 0.01^b^ | 0.04 ± 0.01^b^ | 27.73 ± 0.05^b^ | 61.41 ± 0.30^b^ |
| As: arsenic, Cd: cadmium, Cu: copper, Zn: zinc. After 60 days in pots. Values are means ± S.D. (n = 16). Different letters indicate means that are significantly different from each other (one-way ANOVA; LSD test, P < 0.0001). C-: non inoculation, N4: inoculation with *Pseudomonas* sp. N4, N8: inoculation with *Pseudomonas* sp. N8, N10: inoculation with *Ensifer* sp. N10, N12: inoculation with *Ensifer* sp. N12, N4+N10: Co inoculation with *Pseudomonas* sp. N4 and *Ensifer* sp. N10, N8+N10: Co inoculation with *Pseudomonas* sp. N8 And *Ensifer* sp. N10, N4+N12: Co inoculation with *Pseudomonas* sp. N4 and *Ensifer* sp. N12, N8+N12: Co inoculation with *Pseudomonas* sp. N8 And *Ensifer* sp. N12 and Consortium CSN: Co inoculation with *Pseudomonas* sp. N4 , *Pseudomonas* sp*.* N8, *Ensifer* sp. N10 and *Ensifer* sp. N12 | | | | |


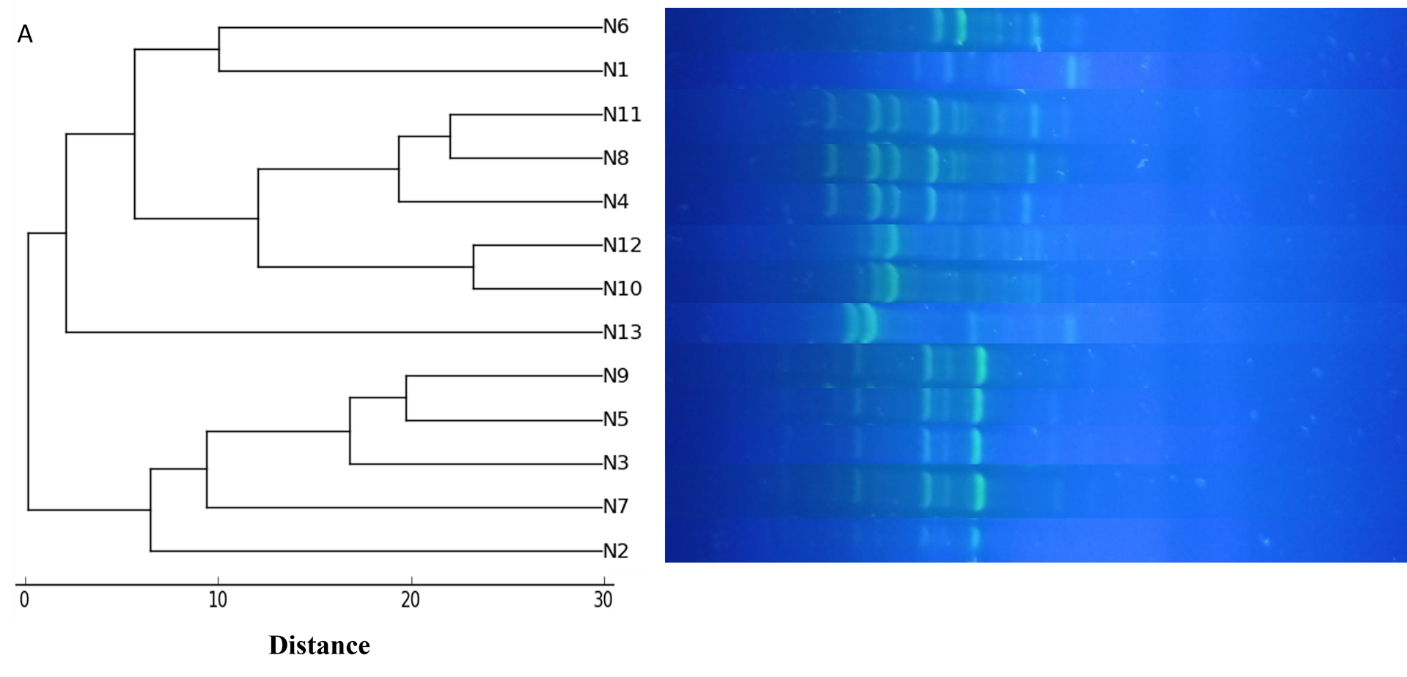


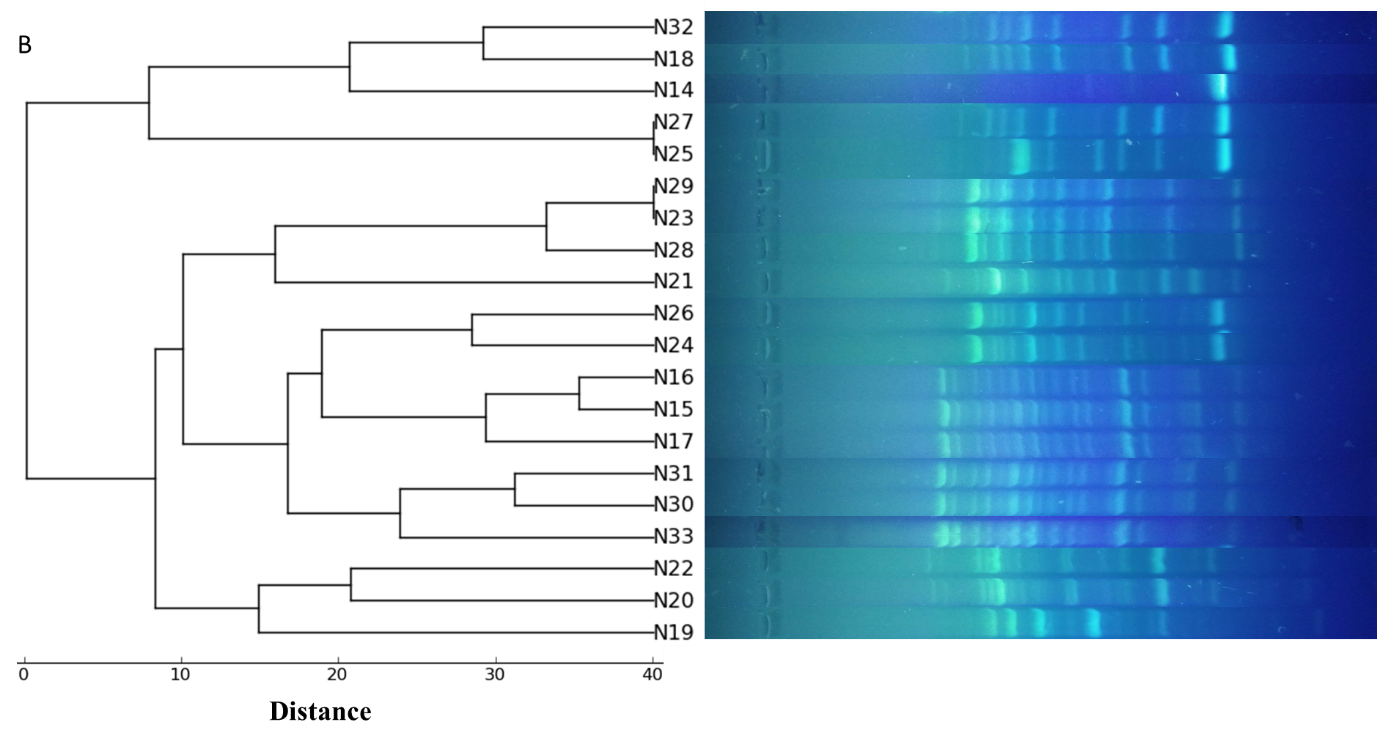


**Supplementary Figure 1**. Box-PCR dendrogram. Diversity of isolates represented in a dendrogram created using UPGMA method. (A) Bacteria isolated in TY medium (Tryptone-Yeast extract agar) and (B)Bacteria isolated in TSA medium (Tryptone Soy Agar)


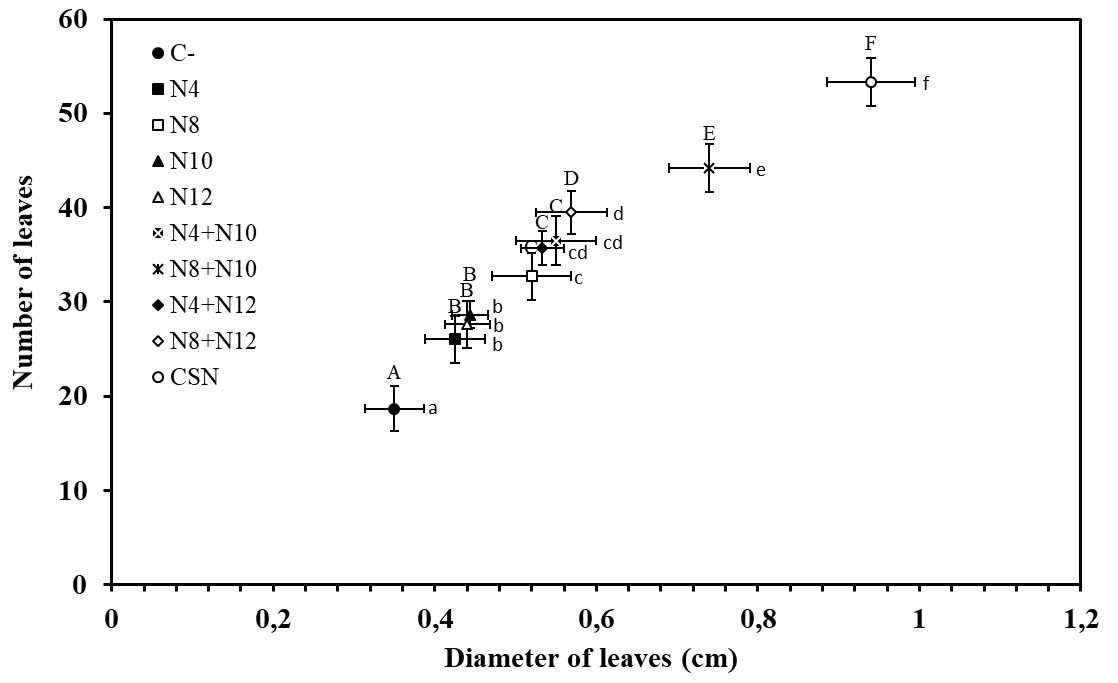


**Supplementary Figure 2. Effects of selected PGPE in *M. sativa* *in pots.*** Number and diameter of leaves in plants of *M. sativa* after 60 days. Soil from the high marshes of the Odiel River. Values are means ± S.D. (*n* = 16). Size of the circles indicates the mean of the diameter of the leaves. Different letters indicate means that are significantly different from each other (one-way ANOVA; LSD test, P < 0.0001) C-: non inoculation, N4: inoculation with *Pseudomonas* sp. N4, N8: inoculation with *Pseudomonas* sp. N8, N10: inoculation with *Ensifer* sp. N10, N12: inoculation with *Ensifer* sp. N12, N4+N10: Co-inoculation with *Pseudomonas* sp. N4 and *Ensifer* sp. N10, N8+N10: Co- inoculation with *Pseudomonas* sp. N8 And *Ensifer* sp. N10, N4+N12: Co-inoculation with *Pseudomonas* sp. N4 and *Ensifer* sp. N12, N8+N12: Co-inoculation with *Pseudomonas* sp. N8 And *Ensifer* sp. N12 and consortium CSN: inoculation with *Pseudomonas* sp. N4 , *Pseudomonas* sp*.* N8, *Ensifer* sp. N10 and *Ensifer* sp. N12 .

**Supplementary Figure 3**. Photosynthetic parameters of *M. sativa* with PGPE in soil from the high marshes of the Odiel River. Electron transport rate. After 60 days in pots. Values are means ± S.D. (*n* = 16). (One-way ANOVA, LSD test, P < 0.0001. C-: non inoculation, N4: inoculation with *Pseudomonas* sp. N4, N8: inoculation with *Pseudomonas* sp. N8, N10: inoculation with *Ensifer* sp. N10, N12: inoculation with *Ensifer* sp. N12, N4+N10: Co-inoculation with *Pseudomonas* sp. N4 and *Ensifer* sp. N10, N8+N10: Co-inoculation with *Pseudomonas* sp. N8 And *Ensifer* sp. N10, N4+N12: Co-inoculation with *Pseudomonas* sp. N4 and *Ensifer* sp. N12, N8+N12: Co-inoculation with *Pseudomonas* sp. N8 And *Ensifer* sp. N12 and consortium CSN: inoculation with *Pseudomonas* sp. N4 , *Pseudomonas* sp*.* N8, *Ensifer* sp. N10 and *Ensifer* sp. N12 .
